# Empagliflozin protects against atherosclerosis progression by modulating lipid profiles and sympathetic activity

**DOI:** 10.1186/s12944-021-01430-y

**Published:** 2021-01-12

**Authors:** Yihai Liu, Jiamin Xu, Mingyue Wu, Biao Xu, Lina Kang

**Affiliations:** 1 Department of Cardiology, Nanjing Drum Tower Hospital, Clinical College of Nanjing Medical University, Jiangsu 210008 Nanjing, China; 2 Department of Cardiology, Affiliated Drum Tower Hospital, Nanjing University Medical School, 210008 Nanjing, Jiangsu China

**Keywords:** Atherosclerosis, Sodium‐glucose cotransporter 2 inhibitor, Empagliflozin, Sympathetic activity, Renin‐angiotensin‐aldosterone system

## Abstract

**Background:**

Several large clinical trials have confirmed the cardioprotective role of sodium-glucose cotransporter 2 inhibitors (SGLT2i) in patients with type 2 diabetes. However, whether empagliflozin, as an SGLT2i, could alleviate atherosclerosis progression in non-diabetic states remain unknown.

**Methods:**

ApoE-/- mice were fed a Western diet for 12 weeks to induce atherosclerosis. On the 7th week, a group of mice were treated with drinking water containing empagliflozin (10 mg/kg/day), while another group was given normal water. At the 12th week, the whole aortas of each group were harvested. Oil Red O, HE and Movat staining were performed for atherosclerotic lesion area and size. Mouse serum lipid profiles (total cholesterol [TC], triglyceride [TG], low-density lipoprotein-c [LDL], and high-density lipoprotein-c [HDL]), systemic inflammation levels (IL-1β, IL-6 and IL-10), renin-angiotensin-aldosterone system (RAAS) components and sympathetic activity (norepinephrine and neuropeptide Y) indicators were measured by ELISA.

**Results:**

Empagliflozin reduced the atherosclerotic lesion burden (-8.6 %, *P* = 0.004) at aortic root in ApoE-/- mice. In addition, empagliflozin decreased body weight (-3.27 g, *P* = 0.002), lipid profiles (TC: [-15.3 mmol/L, *P* = 0.011]; TG: [-2.4 mmol/L, *P* < 0.001]; LDL: [-2.9 mmol/L, *P* = 0.010]), RAAS (renin [-9.3 ng/L, *P* = 0.047]; aldosterone [-16.7 ng/L, *P* < 0.001]) and sympathetic activity (norepinephrine [-8.9 ng/L, *P* = 0.019]; neuropeptide Y [-8.8 ng/L, *P* = 0.002]). However, the anti-inflammatory effect of empagliflozin was not significantly evident.

**Conclusions:**

The early atherosclerotic lesion size was less visible in empagliflozin-treated mice. Empagliflozin could decrease lipid profiles and sympathetic activity in atherosclerosis.

## Background

Sodium-glucose cotransporter 2 (SGLT2) is mainly distributed in the proximal tubule of the kidney and is responsible for reabsorption of 80 %-90 % of glucose load [1]. SGLT2 inhibitors (SGLT2i) can reduce glucose reabsorption in the proximal tubules and increase urine glucose excretion with high selectivity and specificity, thereby reducing blood glucose levels [2]. With the loss of glucose in the urine, body weight and blood pressure also decrease significantly [3].

Recent clinical studies have shown that SGLT2i can reduce cardiovascular mortality and heart failure hospitalization rates in patients with type 2 diabetes [4–6], making them the first hypoglycemic agents to reduce cardiovascular adverse events independent of glycemic control [7]. Some studies revealed that SGLT2i can inhibit inflammation and improve insulin resistance[8], as well as modulate the gut microbiota of type 2 diabetes mice[9]. For nondiabetic mice, SGLT2i may play an antioxidant and anti-inflammatory role [10]. Some studies reported that their cardioprotective role could be associated with reduced arterial stiffness [11], improved myocardial metabolism [12] and increased antioxidative capacity [13]. Ken et al. systematically reviewed preclinical data on the cardioprotective effects of SGLT2i and found that a reduction in atherosclerosis was one of the underlying mechanisms [14].

Atherosclerosis is a systemic pathological process, accompanying fat deposition and chronic inflammation within the artery wall. Atherosclerosis is the leading cause of the majority of clinical cardiovascular events, such as myocardial infarction and stroke [15]. Many risk factors contributed to the initiation and progression of atherosclerosis. Overactivation of the sympathetic system accelerated atherosclerosis while renal denervation mitigated atherosclerosis [16, 17]. Renin-angiotensin-aldosterone system (RAAS) mainly acted on vessels, and promoted the development of hypertension, insulin resistance, vascular and systemic inflammation [18].And RAAS inhibitors have been widely used in atherosclerosis prevention [19]. Previous studies found that SGLT2i could mitigate atherosclerosis by reducing weight and fat, as well as the infiltration of inflammatory cells in plaque [20]. Besides, SGLT2i reduced atherosclerosis by enhancing lipoprotein clearance or decreasing resident macrophages in type 1 diabetic mice [21, 22]. It is well to known that SGLT2i could alleviate the atherosclerosis in diabetic patients. However, the potential role and mechanisms of SGLT2i in atherosclerosis without diabetes are not fully understood.

Therefore, it is hypothesized that empagliflozin, as a kind of SGLT2i, can inhibit the progression of atherosclerosis in a non-diabetic model by virtue of lipid lowering, regulation of sympathetic activity and RAAS.

## Materials and Methods

### Animals

**Fig. 1:**
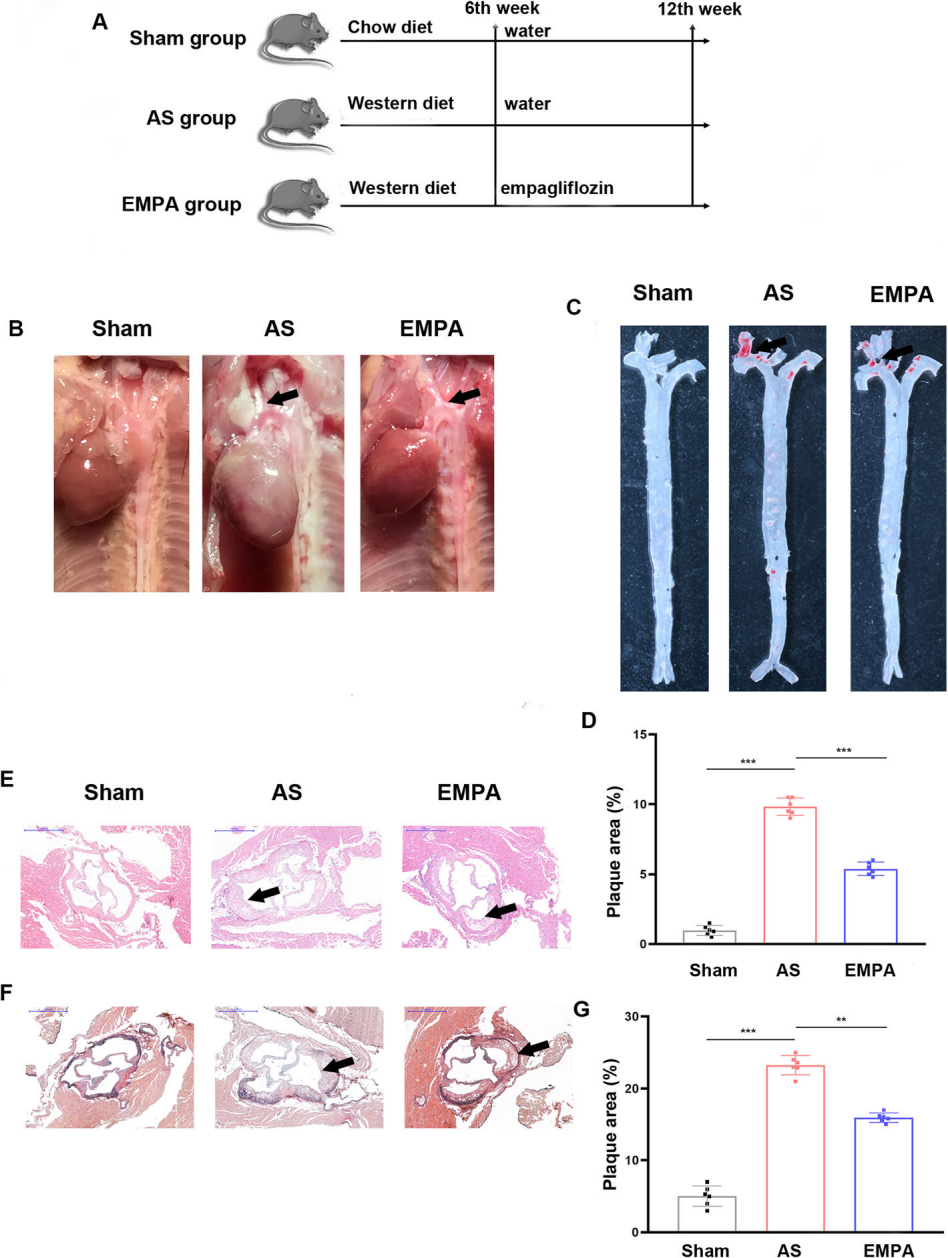
Empagliflozin attenuated the atherosclerotic lesion area. Schematic protocol for mice treatments (**a**). Representative macroscopic image (**b**), En face Oil Red O staining (**c**) and quantitative results (**d**), HE staining (**e**), Movat staining (**f**) and quantitative results (**g**). n = 6, ** P<0.01, ***P<0.001. AS, atherosclerosis group; EMPA, empagliflozin group. One-way analysis of variance with the Bonferroni post hoc test was used. The black arrow indicated the atherosclerotic lesion on aortic arch in **b **& **c**, and on aortic root in **e **& **f**

ApoE-/- male mice (6–8 weeks old, Institute of Model Animals of Nanjing University, Nanjing, China) were kept in a temperature-controlled room (a constant temperature of 23℃ with a humidity of 55–60 %) with a 12-hour light-dark cycle. After 1 week of adaptation to the housing environment, the mice were randomly divided into three groups: sham group, AS group, and EMPA group. The AS group is ApoE-/- mice fed a Western diet containing 0.2 % (wt/wt) cholesterol and 42 % fat (#TP26303, TROPHIC Animal Feed High Tech Co., Ltd, Nantong, China) for 12 weeks. The EMPA group is also fed on a Western diet and received drinking water containing 10 mg/kg/d [23–25] (a higher dose to explore the therapeutic effect on an advanced atherosclerosis model) of empagliflozin (CAS No.: 864070-44-0, MedChemExpress, NJ, USA) beginning at the 7th week. The sham group is ApoE-/- mice fed a chow diet. Each group had 6 mice (Fig. 1A). All mouse studies were approved by the Nanjing University Animal Care and Use Committee. The study was approved by the Ethics Review Board of Nanjing Drum Tower Hospital (No: 2019AE01062).

### Atherosclerotic lesion analysis

After 12 weeks, mice were euthanized with an inhalation overdose of isoflurane and subjected to cervical dislocation. The entire aorta was harvested and observed under a stereomicroscope, and fixed in 4 % paraformaldehyde overnight. Then, the aorta was opened longitudinally and stained in Oil Red O solution (Sevicebio, Wuhan, China) for 2 hours at room temperature. Images were captured using a high-resolution camera. For plaque area analysis in the aortic sinus, the upper portion of the heart above the line connecting the left and right auricles and proximal aorta was fixed and embedded in paraffin. Slides (10 µm) were cut and stained with hematoxylin-eosin or Movat (Sevicebio, Wuhan, China). Images were captured using an Olympus microscope. Lesion size was semi-measured with ImageJ software (NIH, Bethesda, USA), which was not so objective.

### ELISA

Mouse serum was collected at the end of the 12 weeks. The lipid profile [total cholesterol (TC), triglycerides (TG), low-density lipoprotein-c (LDL-C) and high-density lipoprotein-c (HDL-C)], inflammatory cytokines (IL-1β, IL-6 and IL-10), RAAS mediators (renin, angiotensin II and aldosterone) and sympathetic mediators (norepinephrine and neuropeptide Y) were measured by ELISA (Jin Yibai Biological Technology Co., Ltd., Nanjing, China) according to the manufacturer’s instructions using a standard curve (n = 6). The sensitivities for these indexes were 0.16 mmol/L (TC), 0.32 mmol/L (TG), 0.2 mmol/L (LDL-C), 0.2 mmol/L (HDL-C), 5 ng/L (IL-1β), 5 pg/mL (IL-6), 30 pg/mL (IL-10), 5 ng/L (renin), 20 ng/L (AngII), 5 ng/L (ALD), 5 ng/L (NE), and 4.5 ng/L (NPY). The optical densities of the samples were detected using a microplate reader (BIOTEK, Vermont, USA) at a wavelength of 450 nm. All results were shown in Table 1.

**Table 1:** The results of ELISA between Sham, AS and EMPA group

	Sham group, n = 6	AS group, n = 6	EMPA group, n = 6	*P* value
TG	1.56 ± 0.35	4.45 ± 1.18^###^	2.05 ± 0.58^***^	< 0.0001
TC	33.37 ± 11.27	68.66 ± 7.94^##^	53.38 ± 4.28^*^	< 0.0001
HDL	5.31 ± 1.83	8.57 ± 2.32^#^	6.49 ± 1.58	0.0549
LDL	6.97 ± 1.67	15.10 ± 2.05^##^	12.16 ± 0.69^*^	< 0.0001
IL-1β	60.72 ± 1.70	60.95 ± 2.92	59.32 ± 3.64	0.6401
IL-6	72.48 ± 1.81	83.22 ± 5.79	82.47 ± 8.95	0.0327
IL-10	335.2 ± 24.0	426.2 ± 34.63^#^	392.3 ± 27.06^*^	0.0026
Renin	143.4 ± 8.72	156.8 ± 6.24^##^	147.5 ± 3.81^*^	0.0208
Ang II	269.6 ± 33.53	287.3 ± 11.96	305.5 ± 9.38	0.0692
ALD	115.8 ± 2.92	127.9 ± 6.20^##^	111.2 ± 4.23^***^	0.0003
NE	73.77 ± 5.38	86.87 ± 4.29^##^	77.94 ± 3.07^*^	0.0045
NPY	91.82 ± 1.98	98.54 ± 2.62^##^	89.72 ± 3.93^**^	0.0026

#*P* < 0.05, ##*P* < 0.01 and ###*P* < 0.001 vs.. sham; *P < 0.05, **P < 0.01 and ***P < 0.001 vs.. AS

### Statistical analysis

Data are presented as the mean ± standard deviation. One-way analysis of variance with the Bonferroni post hoc test was used for multiple comparisons. For non- parametric variables, Kruskal-Wallis test was used. P < 0.05 was considered statistically significant. All statistical analyses were performed using Prism 8 (GraphPad Software, San Diego, USA).

## Results

### The SGLT2i attenuates atherosclerotic lesion area

To assess the therapeutic role of SGLT2i in atherosclerosis in mice, ApoE-/- mice were fed a Western diet for 12 weeks (AS group), while the EMPA group received empagliflozin at a dose of 10 mg/kg/day from the 7th to the 12th week. The experimental protocol was shown in Fig. 1A. Macroscopically, the atherosclerotic lesion size in the aortic arch was decreased in the EMPA group compared with the AS group (Fig. 1B). En face Oil Red O staining also confirmed the presence of a reduced atherosclerotic lesion area within the aortic arch in the EMPA group (Fig. 1C and D). According to the HE and Movat staining analysis (Fig. 1E, F and G), empagliflozin significantly reduced the lesion size at the cross section of aortic root (-8.6 %, *P* = 0.004).

### The SGLT2i decreases lipid levels in atherosclerosis

**Fig. 2:**
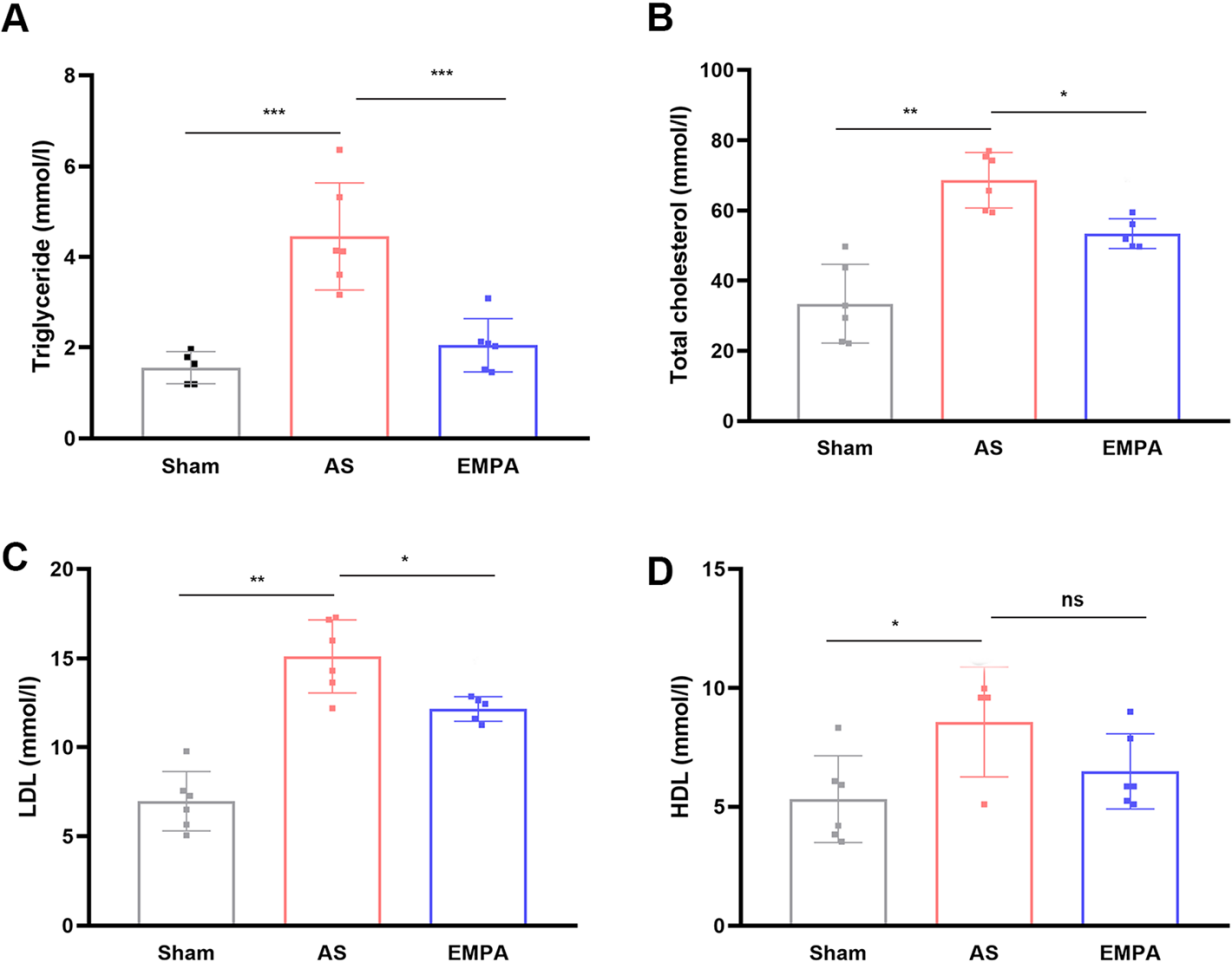
The serum levels of triglycerides (**a**), total cholesterol (**b**), LDL (**c**) and HDL (**d**) in the sham, AS and EMPA groups. AS, atherosclerosis group; EMPA, empagliflozin group. One-way analysis of variance with the Bonferroni post hoc test was used. n = 6, *P<0.05, ** P<0.01, *** P<0.001

Excess lipid deposits contributed to the initiation of atherosclerosis and plaque vulnerability. Therefore, the effect of empagliflozin on lipid profiles of atherosclerotic mice was evaluated. The ELISA results showed that empagliflozin could decrease the levels of triglyceride (2.05 ± 0.58 in the EMPA group vs.. 4.45 ± 1.18 mmol/l in the AS group, *P* < 0.001; Fig. 2A), total cholesterol (53.4 ± 4.28 vs. 68.7 ± 7.94 mmol/l, *P* < 0.05; Fig. 2B), and LDL (12.1 ± 0.69 vs.. 15.1 ± 2.05 mmol/l, *P* < 0.05; Fig. 2C). However, HDL was not significantly different between groups (Fig. 2D).

### The SGLT2i minimally alleviates systemic inflammation in atherosclerosis

**Fig. 3:**
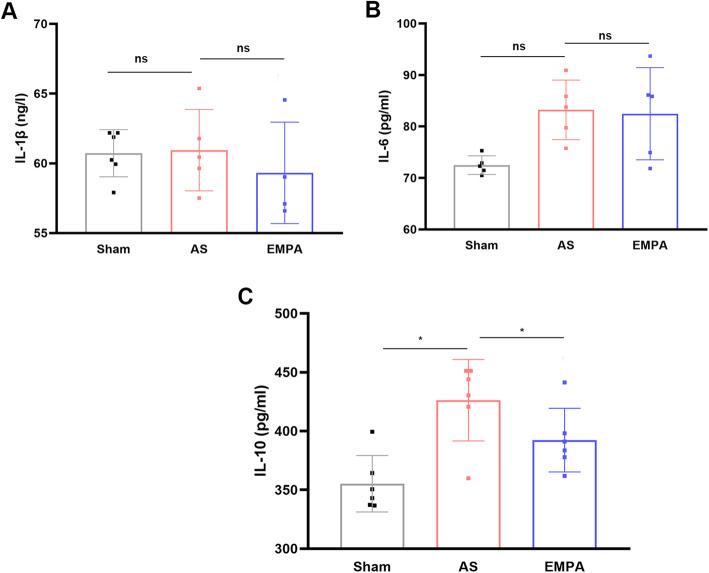
The serum levels of IL-1β (A), IL-6 (B), and IL-10 (C) in the sham, AS and EMPA groups. AS, atherosclerosis group; EMPA, empagliflozin group. One-way analysis of variance with the Bonferroni post hoc test was used. n = 6, *P<0.05, ns, not significant

Chronic inflammation is also an important trigger of atherosclerosis initiation and development. The results found that IL-1β (Fig. 3A) and IL-6 (Fig. 3B) were not significantly decreased in the EMPA group, except for IL-10 (392.3 ± 27.06 pg/ml in the EMPA group vs.. 426.2 ± 34.63 pg/ml in the AS group, *P* < 0.05; Fig. 3C). These results suggested that empagliflozin may have a minimal anti-inflammatory role.

### The SGLT2i inhibits the renin-angiotensin-aldosterone system (RAAS) and sympathetic activity

**Fig. 4:**
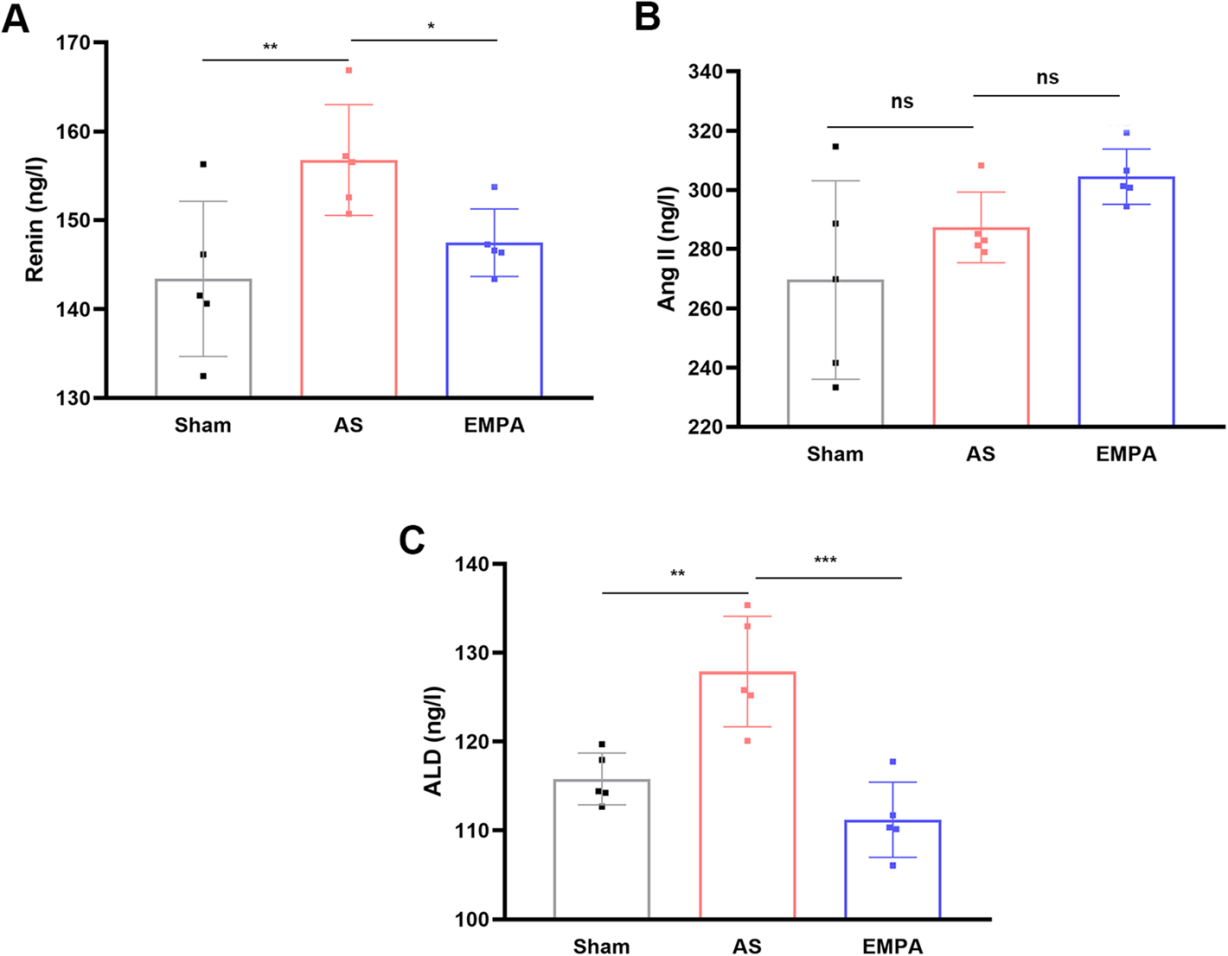
The serum levels of renin (**a**), angiotensin II (**b**) and aldosterone (**c**) between groups. AS, atherosclerosis group; EMPA, empagliflozin group. One-way analysis of variance with the Bonferroni post hoc test was used. n = 6, *P<0.05, ** P<0.01, *** P<0.001, ns, not significant

**Fig. 5:**
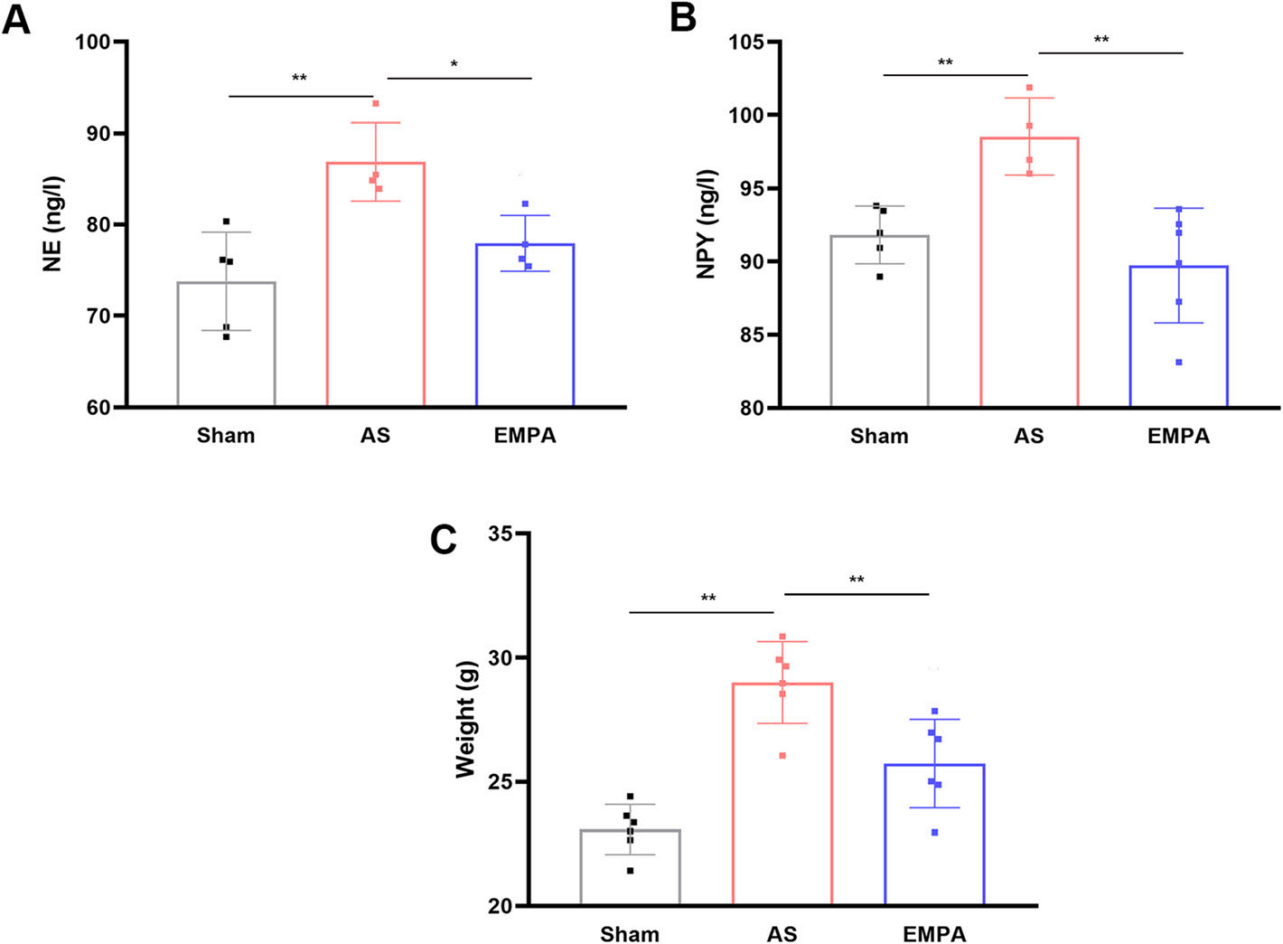
The serum levels of norepinephrine (**a**) and neuropeptide Y (**b**) and body weight (**c**) in the sham, AS and EMPA groups. AS, atherosclerosis group; EMPA, empagliflozin group. One-way analysis of variance with the Bonferroni post hoc test was used. n = 6, *P<0.05, ** P<0.01

Chronic activation of the renin-angiotensin-aldosterone system (RAAS) contributes to vascular remodeling. The results showed that renin (Fig. 4A) and aldosterone (Fig. 4C) were increased in the AS group. While empagliflozin partially inhibited the levels of renin (147.5 ± 3.81 in the EMPA group vs.. 156.8 ± 6.24 ng/l in the AS group, *P* < 0.05) and aldosterone (111.2 ± 4.23 vs.. 127.9 ± 6.20 ng/l, *P* < 0.001). While angiotensin II (Fig. 4B) didn’t show a statistical difference among groups. This result indicated that empagliflozin could alleviate the activation of the RAAS. In addition to RAAS, sympathetic activation also speeds up the progression of atherosclerosis. The results showed that norepinephrine (77.9 ± 3.07 ng/l in the EMPA group vs.. 86.9 ± 4.29 ng/l in the AS group, *P* < 0.05; Fig. 5A) and neuropeptide Y (89.7 ± 3.93 vs. 98.5 ± 2.62 ng/l, *P* < 0.01; Fig. 5B) were partially inhibited after empagliflozin treatment. Interestingly, empagliflozin also decreased the body weight (Fig. 5C) of AS mice (-3.27 g, *P* = 0.002). Considering that SGLT2i could regulate the differentiation of epicardial adipose tissue and perivascular adipose tissue, as well as improve insulin resistance and fat distribution [26]. Therefore, empagliflozin may decrease fat mass induced by a high-fat diet [27, 28].

## Discussion

It is widely acknowledged that ApoE mice fed a high-fat diet can be used to establish an advanced atherosclerosis model [29–32]. The study showed that empagliflozin could reduce atherosclerotic plaques in ApoE-/- mice. At the same time, group of animals actively treated by EMPA was reported with decreased body weight, improved lipid profiles, and reduced RAAS and sympathetic activity, but not with the significant anti-inflammatory effect.

Excessive lipid deposition promotes the development of atherosclerosis. However, the effect of SGLT2i on lipid profiles is not consistent in animal studies. Several previous studies have shown that SGLT2i can lower lipid levels [21, 33–35], while others have not [36–40]. The results showed that empagliflozin could reduce the levels of TC, TG and LDL-C, while there was no significant difference in HDL-C between the two groups. SGLT2i may decrease lipid uptake and metabolism by modulating related genes, such as Mrs1, Scarb1, Cd36, Olr1 or Abca1 [29]. However, further studies are needed to fully elucidate the mechanism of SGLT2i on lipid profiles.

Empagliflozin significantly reduced the expression of norepinephrine and neuropeptide Y, as well as renin, and aldosterone. However, angiotensin II was not statistically changed by empagliflozin treatment, which could be supplemented from other sources. The urine Ang II and angiotensinogen levels should be measured instead from the previous study [41]. As an antidiabetic drug, SGLT2i has been confirmed to decrease the risk of cardiovascular death or hospitalization for heart failure, regardless of the presence or absence of diabetes [42]. Previous studies have also demonstrated that SGLT2i reduces the development of atherosclerotic lesions in diabetic and nondiabetic mice [21, 22, 43, 44]. In accordance with previous results, the study confirmed that lipid-lowering and inhibition of sympathetic activity and RAAS contributed to the antiatherogenic effects of empagliflozin.

In addition to lipid deposition and overaction of sympathetic system and RAAS, extensive observations supported the importance of inflammation in atherosclerosis [45]. Some inflammatory cells and mediators promoted the development of atherosclerosis through activating both innate and adaptive immune pathways [46]. Bedsides, a few anti-inflammatory treatments have reduced the risk of atherosclerotic heart disease [47]. However, the systemic inflammation level of atherosclerosis in the SGLT2i group was not significantly different. Two factors can explain this difference: on the one hand, the nondiabetic ApoE-/- mice may not have a significant vascular inflammatory response induced by hyperglycemia. Nakatsu et al. demonstrated that hyperglycemia rapidly induced a vascular inflammatory response, which can be normalized by short-term (7 days) treatment with the SGLT2i luseogliflozin [37]. A previous study confirmed that empagliflozin did not lead to a significant difference in glucose levels in nondiabetic states [48]. On the other hand, the systemic inflammation level is likely affected by the duration of treatment with SGLT2i. The duration in the experiment was 12 weeks, and the experimental group was treated with SGLT2i beginning at the 7th week. Combining previous studies, SGLT2i may inhibit inflammatory mediators with a duration of at least 8 weeks [8, 9, 44, 49]. Therefore, short-term empagliflozin treatment may not be enough to have an anti-inflammatory effect.

In recent years, some mechanisms underlying the beneficial effect of SGLT2i on atherosclerosis have been proposed. In diabetic states, SGLT2 inhibitions might enhance glycemic control and lipoprotein clearance [21] while lowering sympathetic activation [50]. The decreased toxicity of glucose to endothelial cells may be a potential mechanism involved in the prevention of atherosclerosis in diabetic ApoE-/- mice [49]. While in non-diabetic conditions, SGLT2 inhibitors could increase adiponectin levels and reduce fat deposition [20]. Besides, Tracey et al. found SGLT2 inhibitors attenuated human vascular endothelial cell activation and induced vasorelaxation to inhibit atherogenesis [51]. In addition, SGLT2i could enhance lipoprotein clearance through heparan sulfate proteoglycans (HSPGs) and bile acid pathways [21], which could protect against atherosclerosis progression. Besides, vWF, involved in platelet adhesion and aggregation, could be a target of SGT2i [52], however, it needs further experiment validation.

### Study strength and limitations

Overall, SGLT2i had a beneficial effect on the progression of atherosclerosis, partially explaining its cardioprotective effect. Due to a relatively short-term treatment, there may be no significant difference in some experimental results. Future long-term empagliflozin treatment studies should be performed. Besides, this study was only an unregistered animal experiment, and further clinical trials should be conducted for its application in atherosclerosis.

## Conclusions

In summary, the SGLT2i, empagliflozin, may exert a protective role in atherosclerosis by reducing lipid levels and inhibiting sympathetic and RAAS activity. This study had laid a foundation that SGLT2i could be applied for the prevention and treatment of atherosclerosis in the clinical practice.

## Data Availability

The data are available upon request.

## Abbreviations

SGLT2i: Sodium-glucose cotransporter 2 inhibitors
RAAS: Renin-angiotensin-aldosterone system
ACS: Acute coronary syndrome
TC: Total cholesterol
TG: Triglycerides
LDL-C: Low-density lipoprotein-c
HDL-C: High-density lipoprotein-c
HSPG: Heparan sulfate proteoglycans
